# Evaluation of mandibular cortical bone ratio on computed tomography images in patients taking bisphosphonates

**DOI:** 10.1186/s40902-018-0153-5

**Published:** 2018-07-05

**Authors:** Chul-Hong Koo, Jae-Hoon Lee

**Affiliations:** Department of Oral and Maxillofacial Surgery, College of Dentistry, Dankook University, 119 Dandae-ro, Dongnam-gu, Cheonan, South Korea

**Keywords:** Bisphosphonate, Medication-related osteonecrosis of the jaw, Mandibular cortical bone ratio

## Abstract

**Background:**

Bisphosphonate (BP) has the ability to thicken the cortical bone. In addition, it has been reported that the cortical bone thickened by BP has relation to the medication-related osteonecrosis of the jaw (MRONJ). Therefore, the objective of this article is to analyze the ratio as well as thickness of cortical bone in the mandible using computed tomography (CT) and to evaluate it as the predictive factor of MRONJ.

**Methods:**

The thickness of the cortical bone was measured on a paraxial view of the CT showing the mental foramen in 95 patients: 33 patients with MRONJ (3 males, 30 females), 30 patients taking BP without MRONJ (2 males, 28 females), and 32 controls (9 males, 28 females). Also, the ratios of the cortical bone to the total bone were obtained using the measured values. Based on these results, we compared the difference of mandibular cortical bone ratio between the three groups.

**Results:**

The average cortical bone thickness was measured as 3.81 mm in patients with MRONJ, 3.39 mm in patients taking BP without MRONJ, and 3.23 mm in controls. There was only a significant difference between patients with MRONJ and controls (*P* < 0.05). On the other hand, the average mandibular cortical bone ratio was measured as 37.9% in patients with MRONJ, 27.9% in patients taking BP without MRONJ, and 23.3% in controls. There was a significant difference between all groups (*P* < 0.05).

**Conclusion:**

The mandibular cortical bone ratio is large in order of patients with MRONJ, patients taking BP without MRONJ, and controls. This result suggests that the mandibular cortical bone ratio would be very useful to predict the development of MRONJ.

## Background

Bisphosphonate (BP) is widely used in osteoporosis patients [1]. BP is strongly attached to bone matrix and has the function of inhibiting the action of osteoclast [2]. As a result, BP reduces osteoclast action and induces osteoclast apoptosis, thereby reducing bone remodeling. This takes a long time to complete secondary mineralization in existing bone matrix [3–5]. If secondary mineralization persists, the bone matrix density increases and then the cortical bone appears to thicken. This results in increased cortical bone thickness and reduced cancellous bone area [6].

In fact, it was reported that the cortical bone thickness of the femur increased by 1.82% in patients taking alendronate, one of the BP drugs, while it decreased by 0.31% in the control group [7]. In addition, the cortical bone of the second metacarpal bone of patients taking BP was thicker than that of patients not taking BP [8]. Similarly, the cortical bone of the mandible in patients taking BP was thicker than that in the normal subjects [9]. Likewise, several studies have reported that the thickness of the cortical bone is increased when BP is administered [7–10]. According to the leading theory of mechanism of MRONJ development, the capillaries within the bones become embedded in the bones as the secondary mineralization continues. Then, the bone becomes avascular. Because of the injury of the gingiva or invasive surgery, this necrotic bone is exposed and healing is not done well. As a result, MRONJ occurs [11]. Recent studies have shown that the thickness of the cortical bone and the incidence of MRONJ are related [12]. In fact, when the thickness of the mandibular cortical bone was measured, the cortical bone of patients with MRONJ was significantly thicker than that of patients taking bisphosphonate without MRONJ and normal subjects [13].

However, the cortical bone thickness itself may be different depending on the individual characteristics [14]. In addition, the thickness of the cortical bone may change according to the intracortical remodeling between the cortical bone and the cancellous bone [15]. Therefore, the aim of this article is to analyze the ratio as well as the thickness of the mandibular cortical bone using the CT and to evaluate it as the predictive factor of MRONJ.

## Methods

### Study subjects

We studied patients who attended the Department of Oral and Maxillofacial Surgery of Dankook University Dental Hospital in South Korea between February 2012 and September 2017. A total of 95 patients were classified into three groups according to the presence of MRONJ or taking BP. The first group was classified into patients with MRONJ (group A), the second group was patients taking BP without MRONJ (group B), and the last group was patients not taking BP (group C). The following cases were excluded: patients under 65 years of age, with non-menopause, without CT of the entire mandible, with radiotherapy, with osteomyelitis in the mandible, or with oral lesion involving cortical bone in the paraxial view of measurement site. Also, the overall medical history including the age, sex, and the use of BP was examined. In patient taking BP, the administration route and administration period were further investigated. In addition, the MRONJ staging system was also investigated according to the American Association of Oral and Maxillofacial Surgery criteria.

### Study methods

#### CT

CT radiographs taken with the PHT-60FO (VATECH Corp., Hwa-Sung, Korea) were reconstructed into a paraxial view using Pacsplus viewer 3.2 (Pacsplus, Orange, CA, USA).

#### CT analysis

**Fig. 1:**
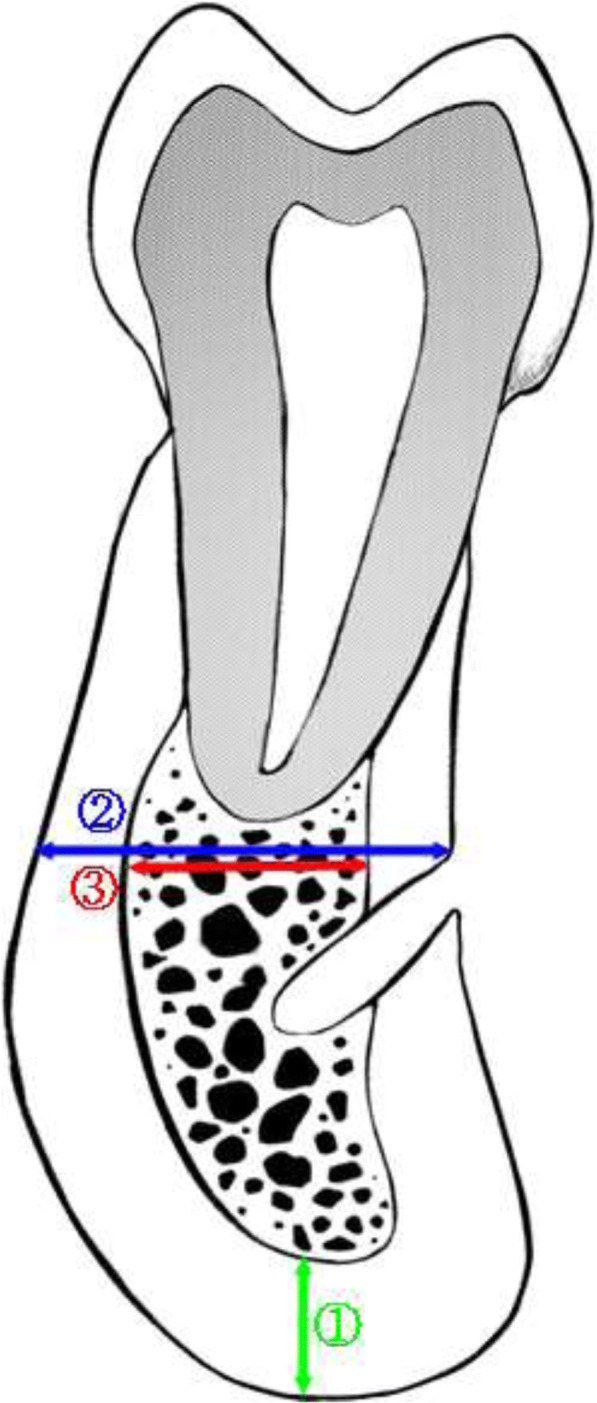
Measurement in paraxial view of CT

Both sides of the mental foramen in the paraxial view of the CT image were used for one patient. Next, thickness of cortical bone was measured at three parts in each section (Fig. 1).

For cortical bone thickness analysis:

- ➀ Thickness of the cortical bone in mandibular inferior border

For cortical bone ratio analysis:

- ➁ Thickness of the total bone on the line parallel to the occlusal plane
- ➂ Thickness of the cancellous bone thickness on the line parallel to the occlusal plane

Using the measured values, the mandibular cortical bone ratio was calculated. First, the thickness of the cortical bone is obtained by subtracting the thickness of the cancellous bone from that of the total bone. Then, mandibular cortical bone ratio was calculated as the percentage of thickness of cortical bone to that of total bone by following formula.

$$ \mathrm{Mandibular}\kern0.5em \mathrm{cortical}\kern0.5em \mathrm{bone}\kern0.5em \mathrm{ratio}\kern0.5em \left(\%\right)=\frac{\textcircled{2}-\textcircled{3}}{\textcircled{2}}\times 100 $$

### Evaluation method

The mandibular inferior cortical bone thickness was compared among the three groups of patients (those with MRONJ, those taking BP without MRONJ, and controls). Next, mandibular cortical bone ratio was compared among the three groups. In addition, correlation between cortical bone ratio and age was confirmed in each group. Lastly, mandibular cortical bone ratio was compared according to the administration period of BP in two groups (those with MRONJ and those taking BP without MRONJ).

### Statistical analysis

For comparative analyses of parametric variables in the three groups, one-way analysis of variance (ANOVA) was performed, followed by the Tukey test to validate the results. For comparative analyses of non-parametric variables in the three groups, Kruskal-Wallis test was performed, followed by the Mann-Whitney test to validate the results. In order to examine the correlation between the two variables, Pearson’s correlation coefficient was obtained. When the variables did not follow a standard distribution, the mean of the two groups divided by administration period was compared using the Mann-Whitney test. IBM SPSS 21.0 software (IBM Co., Armonk. NY. USA) was used to perform the statistical analyses. Differences were considered statistically significant if the confidence interval *P* value was less than 0.05.

## Result

CT images of the patients in the three groups (those with MRONJ, those taking bisphosphonate without MRONJ, and controls) were compared and analyzed to obtain the following results.

### Patient groups

Of the total 95 patients, 81 were women and 14 were men. The mean age was 74.5 ± 5.6 years (Table 1). Group A had a mean age of 74.5 ± 6.0 years, with 30 women and 3 men among 33 patients. Of these, 15 had a history of BP taking more than 4 years and 10 had less than 4 years. Eight could not remember accurately how long they took BP. In addition, 8 had DM, and 25 did not have DM. According to MRONJ staging, stage 1 was 1, stage 2 was 27, and stage 3 was 5.

**Table 1:** Gender, age, administration period, DM, and MRONJ staging of each group

		Group A (*n* = 33)	Group B (*n* = 30)	Group C (*n* = 32)
Gender	Male	3	2	9
	Female	30	28	23
DM	Presence	8	4	8
	Absence	25	26	24
Age	Years (mean ± SD)	74.5 ± 6.0	74.3 ± 5.8	74.8 ± 5.1
	65 ≤ years < 75 (*n*)	21	15	17
	75 ≤ years < 85 (*n*)	11	14	15
	85 ≤ years (*n*)	1	1	0
Administration period (*n* = 49)	Years (mean ± SD)	6.3 ± 5.3	4.8 ± 4.4	–
	< 4 years (*n*)	10	12	–
	≥ 4 years (*n*)	15	12	–
MRONJ Staging	Stage 1	1	–	–
	Stage 2	27	–	–
	Stage 3	5	–	–

A: patients with MRONJ

B: patients taking BP without MRONJ

C: controls

Group B had a mean age of 74.3 ± 5.8 years, with 28 women and 2 men among 30 patients. Of these, 12 had more than 4 years of BP taking, and 12 had less than 4 years. Six could not remember accurately how long they took BP. There were 4 people with DM and 24 people without DM.

Group C had a mean age of 74.8 ± 5.1 years, with 23 women and 9 men among 32 patients. There were 8 people with DM and 24 people without DM.

### Comparison of mandibular inferior cortical bone thickness

The average cortical bone thickness was measured as 3.81 mm in group A, 3.39 mm in group B, and 3.23 mm in group C. There was a significant difference between group A and group C when comparing each cortical bone thickness (*P* < 0.05). On the other hand, there was no significant difference between group A and group B and between group B and group C (Table 2).

**Table 2:** Mandibular cortical bone thickness in each group

	Group	Mean	SD	Significance		
				A–B	A–C	B–C
Cortical bone thickness (mm)	A	3.81	± 0.89	0.211	0.028*	0.863
	B	3.39	± 0.96			
	C	3.23	± 0.86			

A: patients with MRONJ

B: patients taking BP without MRONJ

C: controls

*Significant at the level of *P* < 0.05

### Comparison of mandibular cortical bone ratio

The average mandibular cortical bone ratio was measured at the mental foramen, which was 37.9% in group A, 27.9% in group B, and 23.3% in group C. There was a significant difference between all groups when each cortical bone ratio was compared (*P* < 0.05), (Table 3).

**Table 3:** Mandibular cortical bone ratio in each group

	Group	Mean	SD	Significance		
				A–B	A–C	B–C
Cortical bone ratio (%)	A	37.9	± 7.1	0.000*	0.000*	0.006*
	B	27.9	± 5.4			
	C	23.3	± 4.1			

A: patients with MRONJ

B: patients taking BP without MRONJ

C: controls

*Significant at the level of *P* < 0.05

#### Correlation between cortical bone ratio and diabetes mellitus

The cortical bone ratio between the groups was compared according to the presence or absence of diabetes mellitus (DM). In the groups with DM, there was a significant difference between group A and group C and between group B and group C (*P* < 0.05). But there was no significant difference between group A and group B (Table 4). On the other hand, there was a significant difference between all groups without DM (*P* < 0.05) (Table 5).

**Table 4:** Mandibular cortical bone ratio in each group with DM

	Group	Mean	SD	Significance		
				A–B	A–C	B–C
Cortical bone ratio (%)	A	36.0	± 6.8	0.683	0.002*	0.016*
	B	33.5	± 3.6			
	C	24.1	± 4.7			

A: patients with MRONJ

B: patients taking BP without MRONJ

C: controls

*Significant at the level of *P* < 0.05

**Table 5:** Mandibular cortical bone ratio in each group without DM

	Group	Mean	SD	Significance		
				A–B	A–C	B–C
Cortical bone ratio (%)	A	38.5	± 7.3	0.000*	0.000*	0.006*
	B	27.0	± 5.2			
	C	23.0	± 3.9			

A: patients with MRONJ

B: patients taking BP without MRONJ

C: controls

*Significant at the level of *P* < 0.05

#### Correlation between cortical bone ratio and age

As a result of analyzing the correlation cortical bone ratio and age, there was no significant correlation between the cortical bone ratio and age in each group (Table 6).

**Table 6:** Correlation between mandibular cortical bone ratio and age in each group

Group	Pearson	
	Correlation coefficient	Significance
A	− 0.294	0.097
B	− 0.337	0.069
C	− 0.247	0.174

A: patients with MRONJ

B: patients taking BP without MRONJ

C: controls

*Significant at the level of *P* < 0.05

#### Comparison of cortical bone ratio by administration period

Patients who did not know accurately their administration period were excluded in this comparison. As a result of analyzing the cortical bone ratio by administration period, there was no significant difference in each group (Table 7).

**Table 7:** Comparison of cortical bone ratio by administration period in A and B group

Group	Administration period	Mean	SD	Significance
A	< 4 years	37.9	7.0	0.807
	≥ 4 years	36.6	6.7	
B	< 4 years	26.6	4.5	0.630
	≥ 4 years	28.8	5.9	

A: patients with MRONJ

B: patients taking BP without MRONJ

*Significant at the level of *P* < 0.05

## Discussion

Several studies have observed radiologic features of patients taking BP. It has been reported that the use of bone scan can detect symptom-free osteonecrosis in patients treated with BP and the extent and characteristics of osteolytic lesions can be determined using CT and MRI [16]. Rocha et al. [17] maintained that the change of bone was early confirmed by panoramic radiographs making it easy to diagnose MRONJ. In many kinds of radiological examinations including panoramic radiographs, periapical radiographs, CT, and MRI, it was observed that osteolysis, bone deposition, thickening of lamina dura, and cortical margins when taking BP [18]. In addition, it was reported that patients taking BP showed cortical bone erosion, sequestrum formation, or pathological fracture of the jaw in panoramic radiographs [19].

Moreover, there have been studies quantitatively evaluating changes of the mandible associated with BP on radiographs. Torres et al. [12, 13] compared the thickness, cross-sectional area, and volume of the mandibular cortical bone with MRONJ patients and normal subjects who did not take BP. As a result, MRONJ patients showed larger values ​than normal subjects. Also, they compared the thickness of the mandibular inferior cortical bone in panoramic radiographs among patients with MRONJ, patients taking bisphosphonate without MRONJ, and normal subjects. The thickness of the cortical bone was significantly thicker in patients with MRONJ (6.81 ± 1.35 mm) than those without MRONJ (5.44 ± 1.09 mm) and normal subjects (4.79 ± 0.85 mm). According to Hamada et al. [20], it was observed that MRONJ patients had thicker cortical bone and higher radiodensity of cancellous bone than normal subjects on CT images. In addition, Iwata et al. [21] measured the thickness of the mandibular buccal and lingual cortical bone in three groups: patients with MRONJ, patients taking osteoporosis medication without MRONJ, and non-osteoporotic group. As a result, they found that the cortical bone of patients with MRONJ was significantly thicker than that of the other groups. But they could not find significant difference between patients taking osteoporosis medicine without MRONJ and normal subjects. On the other hand, according to the measurement of the mandibular cortical bone thickness in our study, there was a significant difference only between MRONJ group and normal group. In other words, it is difficult to distinguish patients taking BP without MRONJ from other groups by cortical bone thickness. Therefore, it is difficult to predict the incidence of MRONJ using only cortical bone thickness on patients taking BP.

It was reported that aging of bone tissue and reduction of bone density begins from 45 years of age and bone loss of the cortical bone occurs mainly from about 65 years old [14]. Also, the cortical bone thickness itself varies depending on individual characteristics, such as the shape of the face, occlusal force, and age [22]. Thus, the size of the jaw and the thickness of the cortical bone can vary from person to person. In addition, Zebaze et al. [15] found that cortical bone thickness can be changed by intracortical remodeling between the cortical bone and cancellous bone. Therefore, we examined not only merely the thickness of the cortical bone but also the ratio of the cortical bone to total bone in this study. The mandibular cortical bone ratio was measured in patients with MRONJ, patients taking BP without MRONJ, and controls, and the difference between these groups was confirmed. As a result, mandibular cortical bone ratio was significantly larger in patients with MRONJ than in patients taking BP without MRONJ and controls. It was also found that the mandibular cortical bone ratio was significantly higher in the patients taking BP without MRONJ than in the controls, that is, the measurement of the mandibular cortical bone ratio showed a significant difference between all groups.

DM can cause bone quality deterioration through microvascular ischemia, endothelial dysfunction, reduced bone remodeling, osteoblast, and osteoclast apoptosis and induce changes in immune cell function and inflammation. Therefore, DM increases the risk of chronic infection and MRONJ [23]. In this study, mandibular cortical bone ratios were compared according to the presence or absence of DM. When cortical bone ratio was examined in patients with DM in each group, there was no significant difference between the patients with MRONJ and the patients taking BP without MRONJ (Table 4). On the other hand, in patients without DM, there was a significant difference between the patients with MRONJ and the patients taking BP without MRONJ (Table 5). In other words, in patients with DM, cortical bone ratio of the group with MRONJ is similar to that of the group taking BP without MRONJ. These results suggest that patients taking BP without MRONJ are more likely to develop MRONJ in the presence of DM.

Additionally, the correlation between the mandibular cortical bone ratio and age was analyzed. As a result, the ratio decreased with increasing age in all groups, but it did not show any significant correlation. This is because the subjects were confined to 65 years or older, not all ages.

The two groups taking BP (those with MRONJ and those taking BP without MRONJ) were subdivided by administration period (the 4 years standard). The reason why they are divided by administration period based on 4 years standard is that the prevalence of MRONJ in patients taking BP treatment for more than 4 years increased significantly to 0.21% [2]. Then, difference of cortical bone ratio by administration period was analyzed. It was resulted that there was no significant difference between patients with MRONJ and patients taking BP without MRONJ. This may be due to the fact that the number of patients who were precisely examined for the administration period in each group was low, and the administration period was not accurate because it depends on the patient’s statements. In addition, the difference of the administration route would have affected the outcome.

In this study, the cortical bone thickness and ratio were measured near the mental foramen, which was measured from the basal bone, not the alveolar bone where the MRONJ is predominant. There are two reasons why reference point was near the mental foramen. First, most MRONJ patients have the alveolar bone that has already invaded cortical bone destruction or has undergone bone resorption due to poor periodontal status. Accordingly, it is difficult to set a reference point for thickness and ratio measurement. Lastly, when osteoporosis medication is taken, it acts systemically and it is difficult to occur specific cortical bone thickness changes only in specific areas of the mandible.

Previous studies have measured cortical bone thickness in relation to MRONJ, but there was no significant difference between patients taking BP without MRONJ and normal subjects. However, in this study, we could also find significant differences between patients taking BP without MRONJ and normal subjects by using mandibular cortical bone ratio. Therefore, if further research is progressed in the future, it may be used as a predictor of the incidence of MRONJ in patients who must have oral surgery including extraction.

## Conclusion

We compared the CT findings of patients with MRONJ, patients taking BP without MRONJ, and controls, and the following results were obtained. In measurement of cortical bone thickness, that of patients with MRONJ (group A) is significantly thicker than that of the controls (group C). However, there is no significant difference between patients with MRONJ (group A) and patients taking BP without MRONJ (group B). And, there is no significant difference between patients taking BP without MRONJ (group B) and controls (group C). On the other hand, in measurement of cortical bone ratio, patients with MRONJ (group A), patients taking BP without MRONJ (group B), and controls (group C) are large in order, and there is a significant difference between all groups.

To conclude, it is considered that “ratio of cortical bone to total bone in mandible” would be one of the good predictive factors for MRONJ in the future.

## Abbreviations

BP: Bisphosphonate
CT: Computed tomography
MRONJ: Medication-related osteonecrosis of the jaw
